# P-1635. Back to School! Improving Pharyngitis Care for School Students

**DOI:** 10.1093/ofid/ofae631.1801

**Published:** 2025-01-29

**Authors:** Dana Bjuro, Lisa Davidson, Jean Davison, Carol F Durham, Charles Williams

**Affiliations:** Atrium Health Levine Children's, Charlotte, North Carolina; Atrium Health, Charlotte, North Carolina; UNC - Chapel Hill, NC, Chapel Hill, North Carolina; The University of North Carolina at Chapel Hill, Chapel Hill, North Carolina; Imagine Pediatrics, Charlotte, North Carolina

## Abstract

**Background:**

School-Based Virtual Care (SBVC) clinics in North Carolina (NC) virtually connect pediatric providers with students without the student leaving school. The Centers for Disease Control and Prevention (CDC) encourages organizations to use strategies to improve antibiotic stewardship in telehealth settings. This initiative focused on improving the appropriate treatment of Group A Streptococcus (GAS) pharyngitis by developing and deploying evidence-based education about acute pharyngitis care. This education was distributed to SBVC providers and caregivers of students presenting with pharyngitis in an SBVC clinic. The SBVC clinics selected for this initiative have one of NC's highest prevalence of GAS pharyngitis.

**Figure 1.**
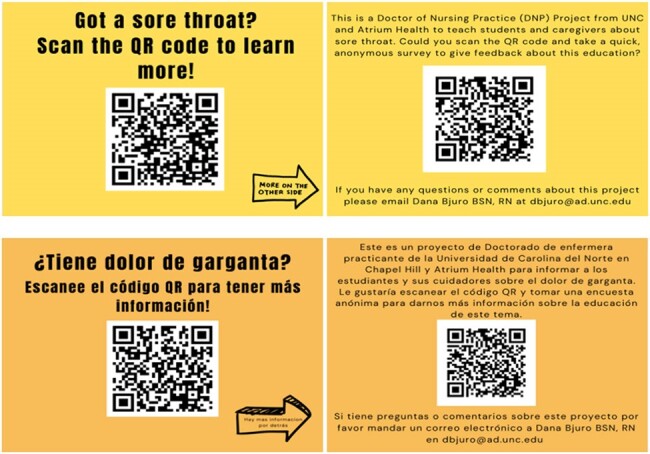
Front and Back of the Educational Postcard Distributed to Caregivers

These postcards were distributed to students with a chief concern of sore throat following their visit with an SBVC provider. Students were asked to take these postcards home to their caregivers. The caregivers were notified by the medical assistant or SBVC provider that the postcard would be sent home with their child. For students testing positive for GAS pharyngitis, a new toothbrush was attached to the postcard to encourage the child to change their toothbrush and to decrease the chance of the postcard being misplaced.

**Methods:**

All SBVC providers in the Pediatric Virtual Care Team received an education module about the organization’s Sore Throat Treatment Algorithm (STTA) and feedback on antibiotic prescribing. For all students presenting with a chief concern of pharyngitis, following their visit with an SBVC provider, the student received a postcard with a link to additional CDC sore throat education for their caregiver to view. If students tested positive for GAS, they received a new toothbrush and the educational postcard. Qualitative data collection through provider and caregiver surveys and quantitative data collection through chart reviews were used to assess the success of this project.

**Figure 2.**
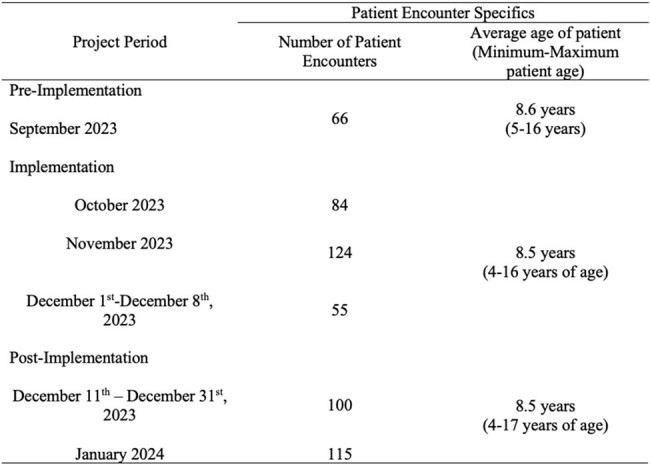
Patient Encounter Specifics During Initiative

This table reflects the number of patient encounters with a chief concern of sore throat and the average age of the patients seen at the specific SBVC clinics with a high prevalence of GAS pharyngitis. Antibiotic prescribing data from patient encounters in September 2023 was used as baseline data prior to the implementation of the initiative.

**Results:**

Following the STTA, manual chart reviews of antibiotic prescribing rates revealed an average decrease in inappropriate antibiotic prescribing by 53.3% following the implementation of evidence-based education for SBVC providers. Sixty-six percent (66%) of all caregivers who completed the post-education survey reported feeling extremely comfortable caring for their child with pharyngitis after viewing the evidence-based education.

**Figure 3.**
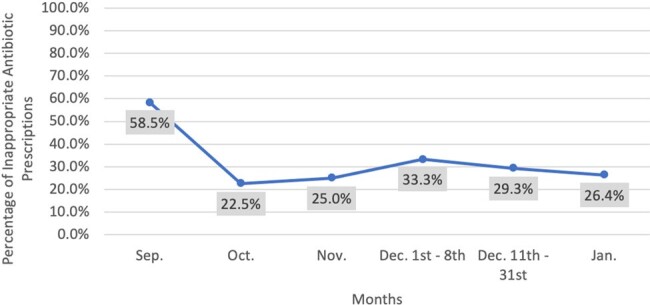
Reduction in Inappropriate Antibiotic Prescribing throughout Implementation Period

The implementation period was from October 2023 through December 8th, 2023. Antibiotic prescribing data was assessed through January 2024 to assess the initiative's sustainability.

**Conclusion:**

Improving adherence to the organization’s STTA and providing caregiver-specific education on pharyngitis has reduced inappropriate antibiotic prescribing and improved caregiver knowledge. Reducing inappropriate antibiotic prescribing is important for students receiving care in these SBVC clinics as it prevents additional illness and mitigates complications from GAS pharyngitis.

**Disclosures:**

**All Authors**: No reported disclosures

